# Splenic Nodular Sclerosis-Variant Hodgkin Lymphoma Presenting as Hemophagocytic Lymphohistiocytosis: A Diagnostic Challenge in an Adult Patient

**DOI:** 10.7759/cureus.107882

**Published:** 2026-04-28

**Authors:** Francisco David Roman Delgado, Oliver Daniel Vasconcelos Prado, Dhavyd Herrera Ibañez, Juan Carlos Anda Garay

**Affiliations:** 1 Internal Medicine, Centro Médico Nacional Siglo XXI of the Instituto Mexicano del Seguro Social (IMSS), Mexico City, MEX

**Keywords:** fever of unknown origin (fuo), hemophagocytic syndrome (hs), hodgkin lymphoma, hyperinflammatory syndrome, lymphohistiocytosis

## Abstract

Hemophagocytic lymphohistiocytosis (HLH), also known as hemophagocytic syndrome, is a life-threatening hyperinflammatory syndrome. Primary HLH is more common in children, while secondary HLH, triggered by malignancy, infection, or autoimmunity, predominates in adults. We report the case of a 56-year-old man referred to our institution with a 10-month history of fever (>38.3°C) meeting diagnostic criteria for fever of unknown origin (FUO). Clinical evaluation revealed splenomegaly and pancytopenia. Given the high clinical suspicion of HLH, an H-score of 219 points was calculated, and management was initiated. As part of the diagnostic workup, Ga-67 citrate scintigraphy and multimodal imaging were performed, revealing intense splenic uptake and structural abnormalities. These findings guided a targeted splenectomy, which confirmed classic splenic Hodgkin lymphoma (HL) (nodular sclerosis subtype). This case illustrates a systematic clinical approach to HLH, enabling the identification of the underlying etiology and facilitating timely intervention. The association between HLH and HL is rare and carries a high mortality rate; however, prompt etiological treatment significantly improves survival. We present, to the best of our knowledge, the first-ever reported case of HLH associated with splenic HL.

## Introduction

Hemophagocytic lymphohistiocytosis (HLH) is a life-threatening hyperinflammatory syndrome characterized by an uncontrolled cytokine storm and multiorgan dysfunction. It is broadly classified into primary HLH (hereditary, typically manifesting in the pediatric population) and secondary HLH (more prevalent in adults with a median age of 50 years old) [1]. Secondary HLH is triggered by diverse underlying conditions, including malignancy, infectious disease, or autoimmune disease [2]. It is a severe, rapidly progressive condition with reported mortality up to 60% [3]. Epidemiological data for adult HLH in Mexico remain limited due to frequent underdiagnosis; a retrospective study from an internal medicine department in Oaxaca, México, reported an annual incidence of 0.16% among hospitalized patients [4].

While the most frequently associated infectious triggers include Epstein-Barr virus (EBV), cytomegalovirus (CMV), human immunodeficiency virus (HIV), and *Mycobacterium *species, malignancies, particularly lymphoid neoplasms, remain the primary cause of HLH in adults [5,6]. 

Among oncologic triggers, Hodgkin lymphoma (HL) is exceptionally rare, representing less than 1% of all malignant neoplasms, with isolated splenic involvement being even rarer. When this malignancy manifests as the driver of HLH, it often presents a significant diagnostic dilemma due to the absence of peripheral lymphadenopathy and its ability to mimic other systemic inflammatory or infectious diseases [7]. 

In this context, we report the case of a 56-year-old male patient, highlighting the diagnostic and therapeutic challenges of HLH secondary to splenic HL, a clinical entity rarely documented in the medical literature.

## Case presentation

A 56-year-old male, originally from Chiapas, Mexico, with no prior medical history, presented with a 10-month history of intermittent fever reaching 40°C. He was referred to our institution from an external facility where several laboratory tests had been performed, including complete blood count, TORCH (toxoplasmosis, rubella, cytomegalovirus, herpes simplex, and HIV) profile, and serologic tests for HIV, hepatitis B virus (HBV), hepatitis C virus (HCV), antinuclear antibodies (ANA), and antineutrophil cytoplasm antibodies (ANCA); all results were unremarkable except for the presence of pancytopenia, characterized by hemoglobin of 6.4 g/dL (reference range 13-18 g/dL), platelets of 80 × 10³/µL (reference range 150-450 × 10³/µL), and neutrophils of 0.9 × 10³/µL (reference range 1.5-7.0 × 10³/µL). A previous bone marrow aspiration led to a presumptive diagnosis of myelodysplastic syndrome (MDS), for which he received different treatments, including prednisone, cyclosporine, and erythropoietin at unspecified doses, an atypical regimen not standard for MDS, without clinical improvement. Due to persistent fever and therapeutic failure, the patient was referred to our institute to initiate a formal diagnostic workup for fever of unknown origin (FUO).

Upon admission, laboratory evaluation revealed significant abnormalities across hematologic and biochemical parameters. The complete blood count demonstrated leukopenia, anemia with normocytic indices and borderline normochromia, as well as severe thrombocytopenia, consistent with a pancytopenic profile. Liver function tests were notable for hyperbilirubinemia, predominantly conjugated, accompanied by a mixed but predominantly cholestatic pattern of liver injury, evidenced by markedly elevated cholestatic enzymes with only mild transaminase elevation. No other clinical evidence of organ failure was identified (Table 1).

**Table 1:** Initial laboratory findings on admission Cholestatic pattern was noted in addition to pancytopenia.

PARAMETER	RESULT	REFERENCE RANGE
Blood Chemistry Panel		
Urea	87.9 mg/dL	16.6-48.5 mg/dL
Creatinine	1.09 mg/dL	0.72-1.25 mg/dL
Total Bilirubin	3.03 mg/dL	0.20-1.20 mg/dL
Direct Bilirubin	2.72 mg/dL	0.00-0.50 mg/dL
Indirect Bilirubin	0.31 mg/dL	0.00-0.50 mg/dL
Alanine Aminotransferase (ALT)	59 U/L	0-55 U/L
Aspartate Aminotransferase (AST)	44 U/L	5-34 U/L
Alkaline Phosphatase (ALP)	760 U/L	40-150 U/L
Lactate Dehydrogenase (LDH)	293 U/L	125-220 U/L
Gamma-glutamyl Transferase (GGT)	1179 U/L	12-64 U/L
Complete Blood Count		
White Blood Cell Count (WBC)	2.74 ×10³/µL	4.60-10.20 ×10³/µL
Neutrophils	0.90 ×10³/µL	1.5-7.0 ×10³/µL
Hemoglobin (Hb)	8.2 g/dL	13-18 g/dL
Mean Corpuscular Volume (MCV)	87.7 fL	80-97 fL
Mean Corpuscular Hemoglobin (MCH)	27.2 pg	27-31pg
Platelet Count	41 ×10³/µL	150-450 ×10³/µL

**Figure 1:**
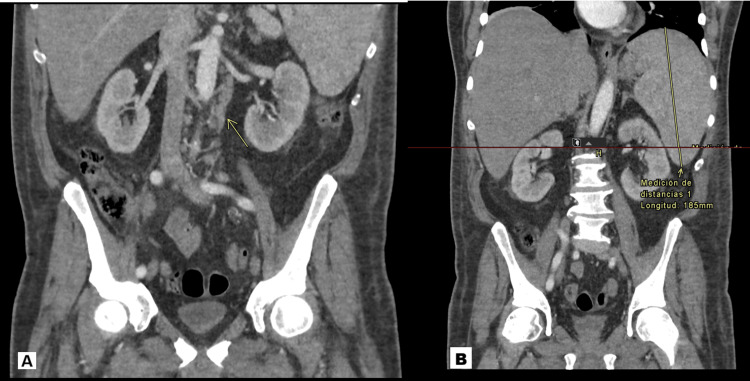
Contrast-enhanced thoracoabdominopelvic computed tomography (CT) A) Extensive infiltrative para-aortic lymphadenopathy, forming nodal conglomerates that suggest vascular encasement or displacement. B) Significant splenomegaly (18.5cm)  with a multinodular pattern, showing multiple poorly defined, diffuse hypodense lesions throughout the splenic parenchyma.

Different tests were done as part of the diagnostic approach for FUO, including a contrast-enhanced thoracoabdominopelvic computed tomography (CT), where splenomegaly with hypodense nodules and retroperitoneal lymph node conglomerates were found (Figure 1).

**Figure 2:**
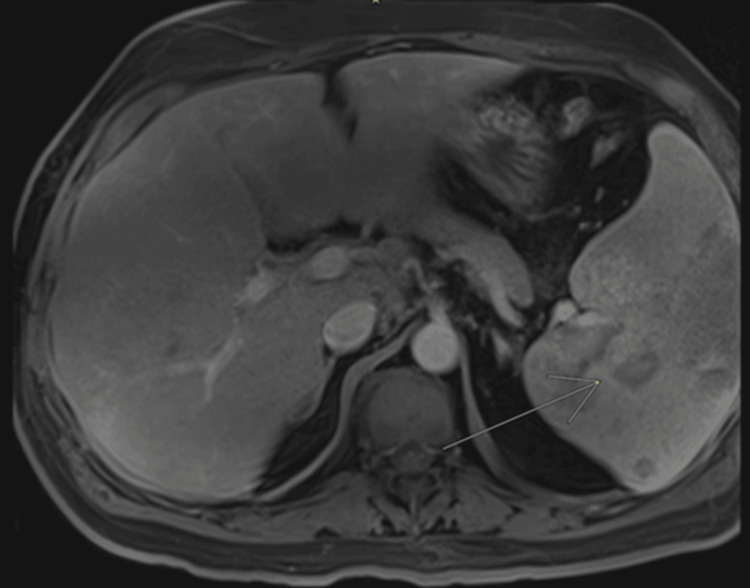
T1-weighted magnetic resonance imaging (MRI) with paramagnetic contrast enhancement. The splenic parenchyma exhibits multiple rounded lesions (yellow arrow) visualized after contrast administration. These lesions are predominantly hypointense at the periphery, featuring diffuse central nodular enhancement with variable diameters measuring up to 38mm. Image suggestive of an infiltrative process.

These findings were further characterized by magnetic resonance imaging (MRI) (Figure 2), suggesting an infiltrative process.

During the diagnostic workup, on the third day of admission, the patient experienced sudden clinical deterioration characterized by progressively worsening cytopenias and persistent fever, so the patient required frequent blood transfusions. In order of clinical evolution, HLH was suspected. Serum triglyceride and ferritin levels were measured, revealing hypertriglyceridemia (215 mg/dL; reference value <150) and marked hyperferritinemia (9,210 ng/mL; reference range 20-300), the latter approaching the extreme levels (>10,000 ng/mL) that are highly suggestive of HLH, along with elevated transaminases. For categorization purposes, a marrow aspiration was performed, which revealed reactive hypocellularity without hemophagocytes; the H-score was calculated at 219 points, indicating a 93%-96% probability of HLH (Table 2).

**Table 2:** The patient's H-Score Calculation and diagnostic probability of hemophagocytic lymphohistiocytosis (HLH)

PARAMETER	PATIENT VALUE	POINTS ASSIGNED
Known Underlying Immunosuppression	No, at the time of calculation	0
Temperature	>39.4°C	49
Organomegaly	Splenomegaly	23
Number of Cytopenias	3 lineages	34
Triglycerides	215 mg/dL	44
Fibrinogen	477 mg/dL	0
Ferritin	9210 ng/mL	50
Aspartate Aminotransferase	38 U/L	19
Hemophagocytosis on Bone Marrow Aspirate	NO	0
Total H-Score	219	
Probability of HLH	93-96%	

Treatment was initiated with immunoglobulin at an initial dose of 2 g/kg divided over three days, and dexamethasone at 10 mg/m² (total immunoglobulin dose: 170 g divided over the three days of treatment and 20 mg/day of dexamethasone, planned for a two-week course), as etoposide, the standard agent per HLH-94 protocol, was not available at our center at the time of admission.

**Figure 3:**
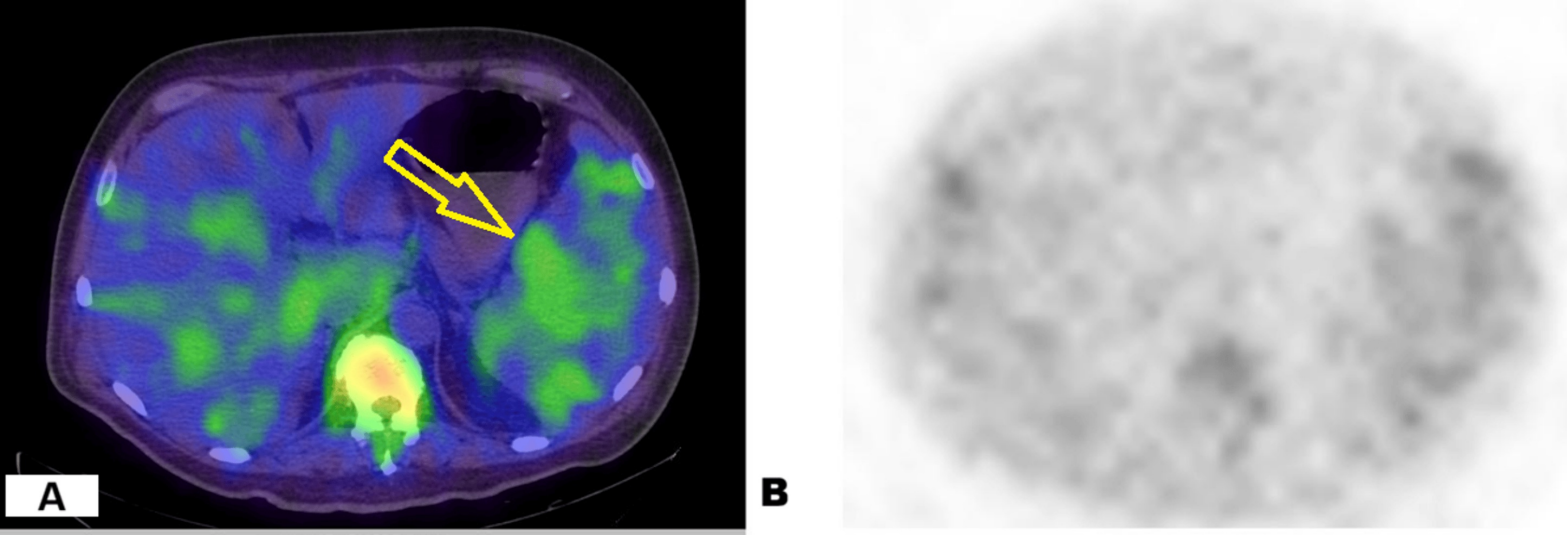
Axial single-photon emission computed tomography/ computed tomography (SPECT/CT) imaging of the abdomen with Ga-67 citrate. A) Axial SPECT/CT fusion: The image demonstrates precise anatomical-functional correlation, showing marked splenomegaly with intense and heterogeneous tracer uptake. The spleen exhibits significantly higher activity than the liver (yellow arrow), confirming an inverted liver-to-spleen functional ratio. Increased vertebral bone activity is also observed, suggestive of marrow expansion or infiltration. B) Axial SPECT image: Functional tracer distribution highlighting prominent splenic and medullary uptake over the background, without superimposed anatomical detail.

Four days after admission, considering a probable lymphoproliferative process as a frequent cause of HLH, a single-photon emission computed tomography/CT (SPECT/CT) scan was performed, revealing increased uptake in the spleen and a retroperitoneal lymph node (Figure 3). 

Given the suspicion of splenic sequestration contributing to thrombocytopenia, splenectomy was performed on hospital day 10 as both a diagnostic and therapeutic intervention (in order to improve platelet consumption and as part of a diagnostic approach looking for lymphoma). Platelet transfusions were administered to maintain counts >50,000/µL perioperatively. The spleen measured 25 x 20 x 15 cm (Figure 3), and it was sent to the pathology department. 

**Figure 4:**
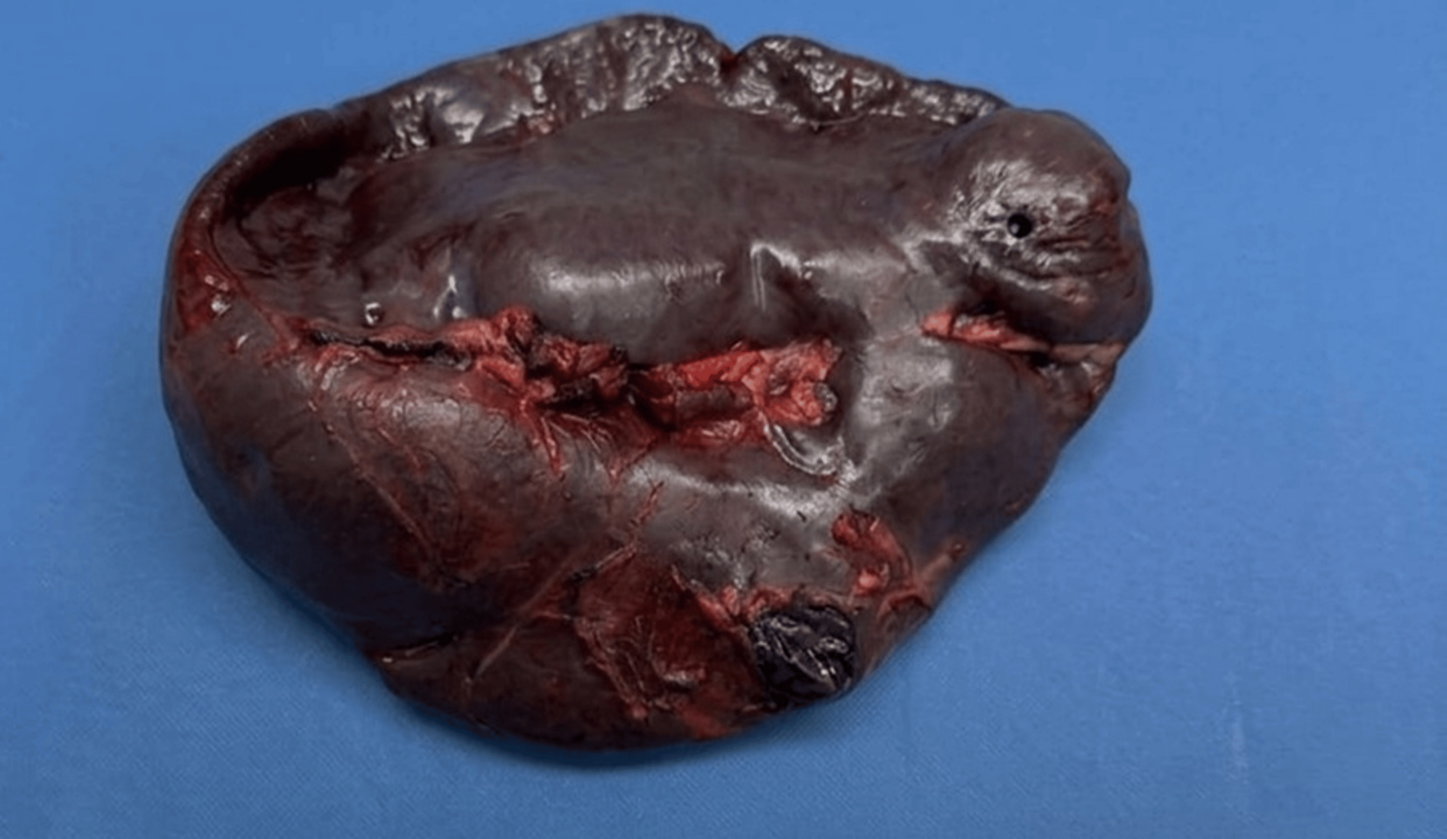
Patient's spleen; dimensions: 25 x 20 x 15 cm, weight 632 grams.

The histopathological report confirmed the diagnosis of HL, a nodular sclerosis subtype; immunohistochemistry was positive for CD30+, CD20+, and CD15+, with a Ki67 proliferation index of 50%.

Following the procedure, the patient showed significant clinical improvement, progressive fever cessation, a progressive increase in platelet counts, and hemoglobin levels. Additionally, a gradual resolution of the hyperinflammatory state was observed based on paraclinical parameters. On hospital day 25, following his postoperative recovery, the patient was referred to a specialized center where chemotherapy was initiated with the ABVD (bleomycin, dacarbazine, doxorubicin, and vinblastine) regimen. The patient experienced treatment failure; ICE (ifosfamide, carboplatin, and etoposide)-nivolumab was given, and the patient presented improvement after three cycles; he is currently asymptomatic.

## Discussion

HLH is typically presented in children, characterized by splenomegaly, cytopenias, hemophagocytosis in bone marrow, and fever (though initial symptoms can be non-specific and diagnostically challenging) [8]; it is mainly precipitated by infectious agents, especially EBV [5]. Our patient presented with the typical characteristics such as splenomegaly, cytopenias, and FUO, but this is a rare case because it is about an adult male with HLH due to a lymphoid neoplasia atypically located in the spleen but with no hemophagocytes found in the bone marrow aspirate, nevertheless making the diagnosis based on H-Score criteria [9], a score system used to predict individual risk for HLH.

Among lymphoproliferative causes of HLH, B-cell non-Hodgkin lymphoma (NHL) is the most common, followed by T-cell NHL; HL is less frequently implicated [10]. This is as in the case of our patient, who represents this minoritarian subgroup. Noyola et al. [11] previously documented a similar case at our institution involving an adult female who presented with HLH secondary to HL diagnosed via submandibular lymph node biopsy; unlike our case, theirs was associated with EBV infection.

Neoplasms are the main cause of HLH in adult patients, lymphomas being responsible for up to 30% of total neoplasms [12]. Although classic HL is described as a potential cause of HLH, the nodular sclerosis variant is uncommon [13,14].

The HLH-94 regimen (etoposide plus dexamethasone) has the best evidence for treating secondary HLH, inducing remission, and significantly reducing mortality [15]. In the HLH-2004 protocol [16], cyclosporin-A was added, but the benefits were uncertain. Rescue therapy options include the L-DEP regimen (PEG-asparaginase, doxorubicin, etoposide, methylprednisolone), primarily studied in EBV-associated HLH, while allogeneic hematopoietic stem cell transplantation is reserved for refractory cases [17]. Currently, emerging therapies targeting specific targets are being described, the impact of which is still under investigation, such as IFN-γ, JAK 1/2 inhibitors, IL-1, IL-6, IL-18, CD52, and PD-1 [18]. Based on everything discussed above and given the evidence shown in the patient's prognosis, we decided to induce remission of the inflammatory response in our patient by administering dexamethasone. Although etoposide is part of the classic treatment regimens, since this resource was not available in our center at the time our patient was hospitalized, we decided to initiate concomitant therapy with immunoglobulin, which has also shown beneficial results in the outcomes of patients suffering from this life-threatening condition [19]. This is exemplified by our case, where prompt pharmacotherapy halted symptom progression until splenectomy removed the primary inflammatory focus. However, it is important to emphasize that in secondary HLH, definitive management requires identification and treatment of the underlying cause. 

Our patient was finally sent to a specialized center focused on oncologic diseases, where they received the ABVD scheme, which is a chemotherapeutic treatment widely used for treating HL (it is worth remembering that this oncologic entity was the cause of the patient’s clinical course). It is documented that the use of this scheme is a definitive treatment in the context of HLH caused by HL; it is also described that the use of other schemes, such as CHOP (cyclophosphamide, doxorubicin, vincristine, and prednisone), rituximab plus CHOEP (cyclophosphamide, doxorubicin, vincristine, etoposide, and prednisone), autologous transplants, and allogeneic hematopoietic stem cell transplantation [10].

Certainly, our patient was sent to our center in order to widely approach an FUO with pancytopenia associated, but given the clinical suspicion of HLH, laboratory studies were amplified, where five out of eight HLH-2004 [16] diagnostic criteria were met. This approach guided the start of immunosuppressive therapy, which had a significant impact on his clinical evolution. An inflammatory focus was documented under lymphoproliferative syndrome suspicion, although active infection by EBV and HIV was not identified, both of which are associated with HLH [15, 20]. EBV immunologic memory could be related to the physiopathology of the case.

This case represents a rare clinical presentation of the splenic HL nodular sclerosis variant, associated with HLH. While HLH is more frequently linked to other histological subtypes, recent evidence highlights the atypical nature of this association in the nodular sclerosis variant [11]. The diagnostic challenge posed by splenic pathology in hematologic disorders is further illustrated by spontaneous splenic rupture in chronic myeloid leukemia, where non-specific abdominal pain may be initially dismissed, leading to fatal outcomes, underscoring the need for high clinical suspicion when splenic involvement is suspected [21].

This case had a favorable outcome thanks to timely management. This type of case should motivate clinicians to carry out a complete approach based on initial clinical suspicion, even in uncommon scenarios. This case contributes to the limited Latin American literature on HLH associated with HL and underscores the importance of a comprehensive and systematic approach that significantly impacts the quality of life of patients.

## Conclusions

HLH is uncommon in adults, but when it occurs, it is predominantly secondary, with neoplasms as the leading etiology. Lymphomas account for one-third of neoplastic causes, with NHL being most frequently implicated. Our patient represents an uncommon clinical case since it was about a lymphoproliferative disease, HL, that presented with HLH, located in an unusual organ, given that it was located in the spleen. These disorders used to be found in lymph nodes. Definitive treatment of secondary HLH lies in control of underlying pathology and remission induction with established schemes, whose evidence shows they are capable of significantly reducing the life-threatening inflammatory response, and, as a consequence, a better prognosis is obtained. Splenectomy is not only therapeutic (for cytopenias), but it is also the definitive diagnostic tool. Better documentation of these types of clinical cases is needed in Latin America and in our country, as it could be the starting point for better epidemiological characterization, better homogenization, and comparison of the clinical presentation of our patients and thus improve the evidence in our environment regarding treatments that have shown a more favorable outcome for our population.
